# Supplements for bone health

**DOI:** 10.20945/2359-4292-2025-0374

**Published:** 2025-12-08

**Authors:** Tiago Donizeti Bertolacini da Silva, Gabriela Mazzarolo Marcondes Vieira, Layana Tyara Sandes Fraga, Wesdrey Dantas Fernandes, Eliane Naomi Sakane, Sergio Setsuo Maeda

**Affiliations:** 1 Departamento de Medicina, Divisão de Endocrinologia, Universidade Federal de São Paulo, São Paulo, SP, Brasil; 2 Departamento de Medicina, Divisão de Clínica Médica, Faculdade de Medicina do ABC, Santo André, SP, Brasil

**Keywords:** Bone health, supplementation, vitamin D, vitamin K, magnesium, phosphorus

## Abstract

Bonehealth is influenced by a dynamic interplay of genetic, hormonal, and environmental factors, with nutrition playing a vital role throughout life. This review consolidates the current evidence on the roles of essential micronutrients, specifically calcium, vitamin D, vitamin K, magnesium, and phosphorus, in skeletal metabolism and integrity. Calcium and vitamin D, the most extensively studied, have been shown to reduce bone loss and fracture risk, particularly in institutionalized individuals or those with deficiencies, while evidence is less consistent in the general population. Although vitamin K, magnesium, and phosphorus are important for bone physiology, the clinical evidence supporting their supplementation is either limited or context dependent. Additionally, this review explores the current status of micronutrient intake in Brazil and discusses potential risks associated with excessive or inappropriate supplementation, such as cardiovascular issues and mineral metabolism disturbances. An individualized, evidence-informed approach may be beneficial in optimizing bone health while minimizing adverse effects.

## INTRODUCTION

Bone health is determined by a complex interplay of genetic, hormonal, and environmental factors, with nutrient intake representing an important contributor to skeletal integrity. Calcium and vitamin D are well-established for maintaining bone mass and reducing fracture risk, particularly in deficient or high-risk populations, whereas evidence supporting the roles of vitamin K, magnesium, and phosphorus is more limited and population specific.

This review has three primary objectives: (^1^) to delineate the physiological functions of calcium, vitamin D, vitamin K, magnesium, and phosphorus in bone metabolism; (^2^) to summarize current dietary intake recommendations and evaluate the Brazilian context; (^3^) to synthesize evidence from meta-analyses and systematic reviews regarding their impact on bone health. Additionally, potential adverse effects related to excessive or inappropriate supplementation are discussed.

## CALCIUM

### Physiological role in bone tissue

Calcium is the most abundant mineral in the human body, playing an essential role in bone formation and regulating various intracellular processes, including cell growth and division, blood coagulation, and muscle contraction (^1^). The majority of the body’s calcium is stored in the skeleton, and daily absorption depends not only on dietary intake but also on physiological mechanisms that regulate calcium distribution and homeostasis. This regulation is mediated through three primary processes: bone resorption, intestinal absorption, and renal reabsorption orchestrated by hormones such as parathyroid hormone (PTH), vitamin D, calcitonin, and fibroblast growth factor 23 (FGF-23). Serum calcium levels, the calcium-sensing receptor, and local regulatory mechanisms in bone, intestine, and kidney ensure calcium availability for physiological functions and skeletal maintenance (^1^).

**Figure 1:**
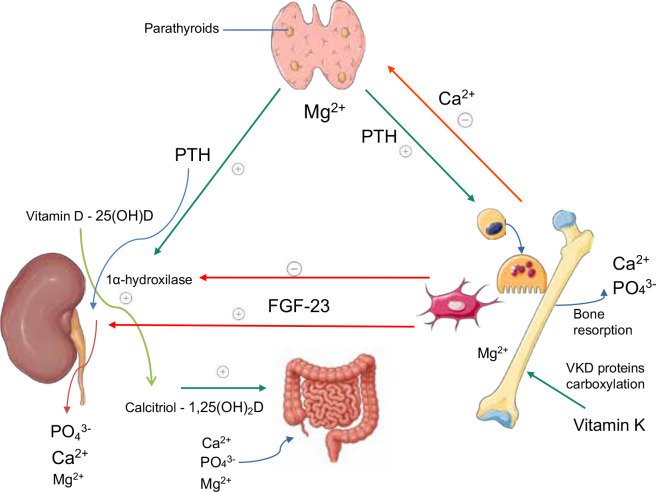
Interactions among the main regulators of mineral metabolism. Parathyroid hormone (PTH), 1,25-dihydroxyvitamin D [1,25(OH)₂D or calcitriol], and fibroblast growth factor 23 (FGF-23) act together to maintain calcium (Ca^2^⁺), phosphate (PO₄^3^⁻), and magnesium (Mg^2^⁺) homeostasis. PTH stimulates bone and renal reabsorption of Ca^2^⁺ and Mg^2^⁺ and induces 1α-hydroxylase, which converts 25(OH)D into calcitriol. Calcitriol, in turn, enhances intestinal absorption of Ca^2^⁺, PO₄^3^⁻, and Mg^2^⁺. FGF-23 decreases renal PO₄^3^⁻ reabsorption and inhibits 1α-hydroxylase activity. Magnesium modulates PTH secretion and action, acts as a cofactor for enzymes involved in vitamin D activation, and contributes to osteoblastic function and bone mineralization. Vitamin K promotes the carboxylation of vitamin K-dependent (VKD) proteins, which are essential for bone mineralization and integrity. This representation was partly generated using Servier Medical Art, provided by Servier, licensed under a Creative Commons Attribution 3.0 unported license.

A decline in serum calcium stimulates PTH secretion, which increases bone resorption and promotes the conversion of vitamin D to its active form, calcitriol, thereby enhancing intestinal calcium absorption and reducing renal excretion. Conversely, elevated serum calcium levels suppress PTH secretion through negative feedback, leading to decreased bone resorption and reduced calcium reabsorption in the kidneys (^1^,^2^). Through these coordinated actions, calcitriol plays a central role in maintaining calcium balance and supporting bone strength. FGF-23, produced by osteocytes and osteoblasts in response to high phosphate, reduces renal phosphate reabsorption and inhibits calcitriol synthesis, indirectly modulating intestinal calcium uptake (^3^). **Figure 1** illustrates the interactions among the main regulators of mineral metabolism.

Within bone tissue, calcium provides structural stability and resistance to mechanical stress while regulating the balance between incorporation into the bone matrix and release into the circulation (^4^,^5^). Bone remodeling, coordinated by osteoblasts, osteocytes, and osteoclasts, ensures daily calcium turnover and maintains skeletal integrity. Insufficient calcium availability can disrupt this balance, leading to bone loss and increased fracture risk, whereas adequate intake supports bone mass maintenance and densification (^5^).

Adequate dietary calcium intake, particularly from dairy sources, is crucial for peak bone mass acquisition during growth and for preserving bone strength throughout life, reducing age-related bone loss and fracture risk (^6^).

### Recommended daily intake of calcium

Humans rely on a daily intake of dietary calcium, which is essential for maintaining physiological functions and skeletal health. Calcium requirements vary depending on the balance between bone formation and resorption, as well as renal and other systemic losses (^1^). Calcium is absorbed throughout the intestinal tract, primarily in the duodenum and jejunum. The fraction absorbed depends on daily intake and is higher when habitual intake is low. During periods of increased demand - such as growth, pregnancy, and lactation - intestinal absorption capacity can increase significantly, reaching up to 60% (^7^). In children and adolescents, calcium retention rises linearly with intake due to both increased absorption and reduced bone resorption; however, absorption declines with aging (^8^).

Calcium absorption occurs via two main mechanisms: (^1^) active transcellular transport in the duodenum and jejunum, and (^2^) paracellular transport throughout the small intestine. The active mechanism plays a particularly important role during low calcium intake, whereas passive diffusion (paracellular transport) predominates with higher intake, accounting for only 8-23% of total absorption. In the duodenum, where vitamin D receptors (VDRs) are abundant, active transport relies on the activation of VDRs and the expression of calcium transport proteins. The active form of vitamin D, 1,25(OH)_2_D, primarily regulates active transport but also influences paracellular absorption (^1^).

Reference values for daily calcium intake vary by age, sex, and life stage. For individuals over 19 years of age, recommendations vary from 1000 to 1300 mg per day, depending on the guidelines adopted (^9^). Many national recommendations align with those of the Institute of Medicine of the National Academy of Sciences (IOM/NAM), which bases intake values on available evidence quality and quantity. For infants (0-6 and 6-12 months), Adequate Intake (AI) reflects average intake in healthy populations due to limited data. From age one onwards, Estimated Average Requirement (EAR) and Recommended Dietary Allowance (RDA) are defined: EAR meets the needs of 50% of healthy individuals, while RDA, derived from EAR, covers nearly all (97-98%) individuals. These values are based on calcium’s effects on bone health (**Table 1**) (^10^).

**Table 1:** Dietary reference values (mg/day) for calcium according to the Institute of Medicine of the National Academy of Sciences (^10^)

Age	AI	EAR	RDA	UL
Infancy to adolescence				
0-6 months	200	-	-	1000
6-12 months	260	-	-	1500
1-3 years		500	700	2500
4-8 years		800	1000	2500
9-18 years		1100	1300	3000
Women				
19-50 years		800	1000	2500
Post-menopause (51+ years)		1000	1200	2000
During pregnancy/lactation (14-18 years)		1100	1300	3000
During pregnancy/lactation (19-50 years)		800	1000	2500
Men				
19-70 years		800	1000	2500
≥ 70 years		1000	1200	2000

AI: adequate intake; EAR: estimated average requirement; RDA: recommended dietary allowance; UL: tolerable upper intake level.

### Sources of calcium

For most individuals, milk and dairy products remain the primary dietary sources of calcium due to their high mineral content and widespread consumption. Alternative sources include dark green leafy vegetables, such as kale, and certain seafood, particularly specific fish species; however, larger quantities of these foods are generally required to meet recommended intake levels (^11^). The Brazilian Food Composition Table, developed by the University of São Paulo, provides detailed data on the calcium content of various food items (**Table 2**) (^12^).

**Table 2:** Calcium content in selected foods (mg/100g)

Category	Food	Calcium (mg/100g)
Dairy products	Parmesan cheese	1050
	Mozzarella cheese	774.55
	Minas cheese (Brazilian fresh cheese)	1020
	Whole milk powder	887.53
	Whole UHT milk	107.74
	Skimmed UHT milk	134.61
	Whole plain yogurt	207.04
Fish/eggs	Canned sardines in oil	393.37
	Cooked salmon	28.76
	Boiled chicken egg	43.72
Vegetables	Raw collard greens	208.43
	Cooked spinach	66.74
	Cooked broccoli	56.83
	Raw watercress	129.77
	Raw arugula	107.28
Legumes	Cooked carioca beans	106.24
	Cooked soybeans	70.81
	Cooked lentils	19
Nuts/seeds	Raw almonds	269
	Raw sesame seeds	825.45
	Raw Brazil nuts	162.63
Processed soy	Tofu	80.76
Cereals/grains	Enriched wheat flour	16.03
	Sliced bread	129.22
	Rolled oats	49.81
Fruits	Banana	221.99
	Orange	29.93
	Strawberry	15.40

Source: Adapted from Brazilian Food Composition Table (version 7.2) (^12^).

### Bioavailability factors

Calcium bioavailability, or the fraction of ingested calcium absorbed by the body, is influenced by several dietary and physiological factors (^13^). Absorption is facilitated by adequate vitamin D levels, gastric acidity, lactose, and non-digestible oligosaccharides (^14^,^15^). Diets high in protein, particularly from animal sources, may enhance fractional calcium absorption, partially compensating for increased urinary calcium excretion (^16^,^17^).

Conversely, several dietary components may reduce calcium bioavailability. Phytic acid, found in fiber-rich foods and cereals, and oxalic acid, present in spinach, chocolate, carrots, sweet potatoes, and cola beverages, are both known to interfere with calcium absorption (^18^). Additionally, agents such as proton pump inhibitors, cholestyramine, tetracycline, and iron supplements may impair calcium bioavailability by reducing intestinal absorption. These agents, therefore, should not be administered concomitantly with calcium (^19^).

Considering both content and bioavailability, milk and dairy products should remain the primary recommended sources of calcium, while alternative sources and supplements can be used when dietary intake is insufficient. Supplementation is generally recommended at 500-600 mg per day, preferably in combination with vitamin D (^20^).

### Calcium supplements

Supplementation is particularly indicated for patients at high risk of calcium and vitamin D deficiency and for those undergoing osteoporosis treatment (^21^). When pharmacological calcium supplements are required, they should be consumed with meals to improve tolerance and enhance absorption (^22^).

Common formulations include calcium carbonate, calcium citrate, calcium citrate malate, and tricalcium phosphate, each with distinct elemental calcium content, absorption characteristics, and clinical considerations (**Table 3**).

**Table 3:** Comparative overview of calcium supplement formulations: elemental content, clinical considerations, and potential adverse effects

Formulation	Elemental Ca (%)	Recommended Use/Dose	Advantages	Potential Limitations/ Adverse Effects
Calcium carbonate	40	Typically 500-600 mg/day, with meals	High elemental calcium content; widely available; cost-effective	Gastrointestinal intolerance; reduced absorption in achlorhydria, atrophic gastritis, or bariatric surgery; may increase kidney stone risk
Calcium citrate	24.1	500-600 mg/day; can be taken with or without food	Well absorbed regardless of food or gastric acidity; safer in achlorhydria or post-gastric surgery	Slightly lower elemental calcium content
Calcium citrate malate	23	500-600 mg/day	Higher bioavailability; reduced GI side effects; minimal interaction with other nutrients; preserves absorption of zinc, magnesium; lower nephrolithiasis risk	Less widely available; moderate elemental calcium
Tricalcium phosphate	37.5	500-600 mg/day; useful for elderly with low dietary phosphorus or intolerance to carbonate/citrate	Moderate elemental calcium; phosphate supplementation	Contraindicated in hyperphosphatemia, chronic kidney disease, or hypoparathyroidism

Source: Adapted from the literature: (^18^,^20^,^23^-^29^).

This comparative overview allows clinicians to select the most appropriate formulation based on patient-specific factors, including tolerance, comorbidities, and dietary intake.

### Brazilian calcium intake scenario

In a population-based study conducted across 150 municipalities in Brazil’s five regions, calcium intake was assessed among men and women aged 40 years and older. The results indicated that residents of the North region had the highest average intake (454 mg/day), irrespective of socioeconomic status. Overall, the average calcium intake for the Brazilian population was approximately 400 mg per day (^30^). However, these estimates may be underestimated due to potential biases inherent in cross-sectional dietary recall methods.

A cross-sectional survey of adolescents aged 15-18 years living in São Paulo revealed that 97% of participants did not meet the recommended daily calcium intake, with an average intake of approximately 700 mg per day (^31^).

Furthermore, an analysis of data from a population survey in São Paulo showed a declining trend in average calcium intake with increasing age, while higher educational levels correlated with greater mineral consumption. The lowest averages were observed among adult women, whereas adolescent males presented the highest values. Despite variations across groups, the most significant finding was the high prevalence of inadequate calcium intake, reaching 85% among adult men and 99% among elderly women (^32^). It is important to note that these results may also be affected by underreporting associated with dietary recall bias.

Lastly, data from a systematic review analyzing studies conducted since 2010 in 74 countries indicate that in Brazil, the average daily calcium intake among adults, both men and women, is approximately 505 mg per day (^33^). These findings reinforce the scenario of inadequate calcium consumption across different regions, age groups, and sexes, suggesting a possible association with the increasing prevalence of osteoporosis in the country.

### Evidence from meta-analyses and systematic reviews

A 2002 meta-analysis evaluated the effect of calcium supplementation on the prevention of osteoporosis in postmenopausal women. This analysis included 15 randomized clinical trials with a total of 1806 participants, comparing calcium supplementation to standard dietary calcium intake. Results indicated that calcium supplementation was more effective than a placebo in reducing bone loss after two or more years of treatment. However, evidence for fracture reduction, particularly concerning non-vertebral fractures, was limited (^34^).

A 2007 meta-analysis by Tang and cols. (^35^) examined the use of calcium supplementation, either alone or in combination with vitamin D, to prevent fractures and bone loss in individuals aged 50 years or older. Findings suggested that supplementation correlated with a 12% reduction in the risk of fractures of all types. Additionally, there was a decrease in the rate of bone mineral density (BMD) loss by 0.54% at the hip and 1.19% at the spine. Daily doses of ≥ 1200 mg of calcium and ≥ 800 IU of vitamin D were linked to better outcomes. Therefore, evidence suggests a modest benefit of calcium use (alone or with vitamin D), particularly in older or deficient individuals, as part of a preventive strategy for osteoporosis in individuals aged ≥ 50 (^35^).

Notably, evidence indicates that calcium from dietary sources may have more favorable effects on bone health in postmenopausal women, possibly due to a more beneficial influence on estrogen metabolism compared to calcium from supplements. This aligns with the notion that calcium’s source may play a crucial role in modulating hormonal effects and maintaining bone health (^36^).

Conversely, a clinical trial involving participants in the Women’s Health Initiative examined the effect of calcium and vitamin D supplementation on fracture risk among postmenopausal women (^24^). The results indicated that supplementation had a more significant impact in specific subgroups, such as women with low calcium and vitamin D intake, older women, and those with osteoporosis or low BMD. These groups experienced a reduced risk of fractures, particularly hip fractures. However, despite the benefits observed in these subgroups, supplementation did not substantially impact fracture risk in the overall study population, highlighting that the effects were confined to women with specific characteristics. An increased risk of kidney stones was also observed (^24^).

Similarly, a longitudinal observational study using data from the Study of Women’s Health Across the Nation found that calcium supplementation was associated with a lower rate of BMD loss at the femoral neck and lumbar spine among middle-aged women, particularly during the premenopausal phase. However, no reduction in fracture risk was observed among supplement users, underscoring the strategy’s limitation for preserving BMD rather than preventing fractures (^37^).

A 2023 systematic review reached a similar conclusion, assessing the effects of calcium and vitamin D supplementation on BMD in premenopausal women. This review included randomized clinical trials comparing calcium, vitamin D, or both with a placebo. Results indicated that supplementation did not lead to clinically significant differences in total hip or lumbar spine BMD compared to placebo. The authors concluded that calcium and vitamin D supplementation, whether alone or combined, is not recommended as a public health intervention to improve BMD in healthy premenopausal women and is unlikely to provide benefits in fracture prevention (^38^).

A recent systematic review suggested that calcium supplementation for fracture prevention is more effective when combined with vitamin D, mainly in reducing hip fractures among elderly and institutionalized individuals at risk of nutritional deficiencies. This combined supplementation produces a synergistic effect on intestinal calcium absorption and reverses secondary hyperparathyroidism, especially when vitamin D levels are below 10 ng/mL (^39^).

### Side effects and adverse events

Hypercalcemia, or elevated serum calcium levels, may occur as an adverse effect of calcium supplementation, particularly when taken in high doses. In such instances, acute toxicity may develop, ranging from mild to severe hypercalcemia. Mild hypercalcemia is often asymptomatic, while moderate hypercalcemia may also be asymptomatic or present with nonspecific symptoms such as decreased appetite, nausea, vomiting, constipation, abdominal pain, dry mouth, and excessive thirst. In cases of severe hypercalcemia, neurological symptoms may emerge, including confusion, delirium, and coma, potentially progressing to death if not properly treated (^40^).

The tolerable upper intake level (UL) establishes thresholds above which nutrient intake may increase the risk of adverse health effects. These values should not be considered recommended intake targets. Excessive calcium intake through a habitual diet is rare and more commonly associated with supplement use (**Table 1**) (^10^).

Calcium supplements, particularly calcium carbonate, are known to cause gastrointestinal side effects such as constipation and flatulence, which are generally regarded as mild discomforts. However, these adverse effects are common and significantly contribute to poor treatment adherence (^41^). A 2019 meta-analysis summarized these symptoms, which include constipation, abdominal cramps, bloating, upper gastrointestinal events, and diarrhea. Furthermore, increased hospital admissions for acute abdominal symptoms were reported in groups treated with calcium, suggesting that these more serious gastrointestinal events may outweigh the benefits of calcium supplementation for fracture prevention (^41^,^42^).

The association between calcium supplements and the risk of nephrolithiasis (kidney stones) remains controversial. Supplement use may increase urinary calcium excretion, thereby elevating the risk of stone formation (^24^,^43^,^44^). However, other studies have found no significant increase in nephrolithiasis among individuals who use calcium supplements (^45^-^47^). Notably, adequate dietary calcium intake has been linked to a lower risk of kidney stone formation, as calcium from food binds to oxalate in the intestine, reducing its absorption and urinary excretion, which are two of the main risk factors for stone formation (^44^,^48^). Therefore, dietary calcium protects against stones, while supplemental calcium may increase the risk of nephrolithiasis.

### Calcium supplementation and cardiovascular risk

A growing body of evidence has examined the potential cardiovascular risks associated with calcium supplementation across diverse populations and study designs. **Table 4** summarizes the main findings from clinical trials, meta-analyses, cohort studies, and guideline statements addressing this issue, highlighting the heterogeneity of results and the influence of factors such as dosage, co-administration with vitamin D, and underlying comorbidities.

**Table 4:** Evidence on cardiovascular risks associated with calcium supplementation

Study Type/ Population	Intervention/ Comparison	Main Findings	Conclusions/Observations	References
Patients with CKD	Calcium supplements	Increased arterial calcification	Suggests potential cardiovascular risk; prompted investigation in other populations	(^49^,^50^)
Randomized placebo-controlled trial (women)	Calcium vs placebo	Higher incidence of MI in the calcium group	Secondary analysis; calcium group had more baseline risk factors (smoking, dyslipidemia, hypertension)	(^51^)
Meta-analysis (15 trials)	Calcium without vitamin D	Higher relative risk of MI	Indicated increased cardiovascular risk, particularly for MI	(^52^)
Women’s Health Initiative calcium and vitamin D trial	Initiation of supplementation during follow-up	Increased MI risk, independent of dose (< 500 mg/day or ≥ 1000 mg/day)	Adverse effect not dose-dependent	(^53^)
Swedish cohort (median follow-up: 19 years)	Dietary and supplemental calcium intake	Nonlinear association between calcium intake and mortality: increased risk with < 600 mg/day and ≥ 1400 mg/day	Higher mortality from ischemic heart disease; greatest risk in women with high dietary intake plus supplements; no association with stroke	(^54^)
Prospective cohort (n = 23,980; mean follow-up: 11 years)	Calcium supplements alone	Increased MI risk	Risk highest among users taking calcium without other micronutrients	(^55^)
Subanalysis of RCT (elderly women)	1200 mg/day calcium vs placebo	No significant difference in CV events or MI	Suggests no increased cardiovascular risk at this dosage	(^56^)
Pooled analysis (older adults)	Calcium + vitamin D vs vitamin D alone	Reduced mortality with combined supplementation	Protective effect observed only with calcium + vitamin D	(^57^)
Critical review of major meta-analyses	Various RCTs	Significant methodological limitations	Trials not designed for CV endpoints; inconsistent dosage, duration, and co-administration with vitamin D	(^58^)
Observational studies	Dietary vs supplemental calcium	Dietary calcium not associated with increased CV risk; supplemental calcium potentially harmful	Adverse cardiovascular effects appear confined to supplemental forms	(^55^,^59^,^60^) (^61^)
Clinical guideline (NOF & ASPC, 2016)	Dietary and supplemental calcium (with/without vitamin D)	No significant increase in CV, cerebrovascular, or mortality risk up to 2000 mg/day	Recommended intake ≤2000 mg/day, consistent with IOM/NAM	(^10^,^62^)
Meta-analysis (2021; 13 RCTs)	Dietary calcium 700-1000 mg/day or 1000 mg/day supplement	An increased risk of cardiovascular and coronary disease by 15% in healthy postmenopausal women.	Reinforces concerns about excessive supplementation	(^63^)
American College of Cardiology (2021)	Proposed mechanisms	Transient serum calcium elevations may activate coagulation, enhance smooth muscle contractility, and promote vascular calcification	Mechanistic rationale for accelerated atherosclerosis	(^64^)
UK prospective cohort (n = 434,374)	Habitual calcium supplement use	Higher incidence of CV events and mortality among diabetics; no association in non-diabetics	Diabetes-related insulin resistance and calcium dysregulation may increase susceptibility to CV outcomes	(^65^)

CKD: chronic kidney disease; MI: myocardial infarction; CV: cardiovascular; RCT: randomized controlled trial; NOF: National Osteoporosis Foundation; ASPC: American Society for Preventive Cardiology; IOM/NAM: Institute of Medicine/National Academy of Medicine; UK: United Kingdom.

Although the current data do not allow for a definitive consensus on the cardiovascular effects of calcium supplementation, the absence of robust, well-powered clinical trials limits more precise conclusions. Nevertheless, based on available findings, it is recommended that calcium intake be prioritized through dietary sources, with supplementation reserved for insufficient dietary intake. Furthermore, high-dose supplementation should be avoided, particularly in individuals at increased cardiovascular risk, such as those with diabetes, where adverse effects appear more pronounced.

## VITAMIN D

### Physiological role in bone tissue

To elucidate the interaction between the vitamin D axis and bone tissue, it is essential to recognize that the principal objective of mineral homeostasis is to maintain serum calcium concentrations within a tightly regulated physiological range. This regulation is of critical importance, as calcium plays a vital role in various physiological processes, including hemostasis, synaptic transmission, and muscle contraction (^66^).

Systemic calcium balance, defined as the difference between dietary calcium intake and daily losses through feces, urine, and sweat, governs this interaction. In states of negative calcium balance, compensatory homeostatic mechanisms are activated to preserve normocalcemia. A key adaptive response is upregulation of 1α-hydroxylase activity, resulting in increased synthesis of 1,25-dihydroxyvitamin D [1,25(OH)_2_D], the biologically active form of vitamin D (**Figure 1**). This secosteroid hormone acts on bone tissue by stimulating osteoclastic bone resorption and inhibiting extracellular matrix mineralization (^67^).

In addition to bone tissue, several other organs integral to calcium homeostasis (e.g., the intestinal epithelium, renal tubular cells, and parathyroid glands) express the VDR. Notably, the effects of vitamin D on bone physiology are primarily mediated through systemic endocrine mechanisms, rather than direct activation of VDRs within bone tissue itself (^68^).

### Positive calcium balance

A positive calcium balance develops when calcium intake is normal or low-normal, provided adequate levels of 1,25-dihydroxyvitamin D [1,25(OH)_2_D] are present. Under these conditions, vitamin D contributes to bone homeostasis predominantly through indirect mechanisms, particularly by enhancing intestinal calcium absorption. Although direct actions of vitamin D on osteogenic cells have been demonstrated in *in vitro* and *in vivo* studies, the precise underlying mechanisms remain incompletely understood (^67^,^68^).

Activation of the VDR in intestinal epithelial cells upregulates genes encoding proteins involved in both pathways of calcium transport, as detailed in the “Calcium” section. With respect to direct effects on bone tissue, VDR expression is most prominent in osteoblasts and osteocytes, although it is also present at lower levels in chondrocytes and osteoclasts (^69^). Evidence supporting the effects of vitamin D on these cells during positive calcium balance derives primarily from transgenic mouse models, in which VDR is either overexpressed or selectively deleted in specific bone cell populations. **Table 5** summarizes the principal findings from these studies.

**Table 5:** Bone-related outcomes in transgenic mouse models with cell-specific VDR gene modifications

VDR modification	Target cell type	Bone outcomes
Deletion	Osteoblast precursors	No changes in serum markers (calcium, PTH, phosphate, 1,25(OH)_2_D) or trabecular bone mass
Deletion	Immature osteoblasts	Increased trabecular bone mass due to reduced RANKL production
Deletion	Mature osteoblasts and osteocytes	No differences in trabecular or cortical bone mass compared to the control group
Overexpression	Mature osteoblasts	Increased cortical and trabecular bone mass
Deletion	Osteoclast precursors	No changes in bone metabolism

Source: adapted from literature (^70^-^73^).

### Negative calcium balance

Negative calcium balance occurs when intestinal calcium absorption is insufficient to meet physiological demands. This condition may result from decreased VDR activity or may develop even when VDR expression is normal. Severe vitamin D deficiency and vitamin D-dependent rickets or osteomalacia are disorders associated with diminished VDR-mediated activity. In such conditions, the synthesis of all components essential for intestinal calcium absorption is impaired (^67^,^74^).

Proper VDR expressions in the distal nephron, along with PTH activity at this site, is critical for adequate renal calcium reabsorption. This process closely resembles the active transcellular calcium transport found in the intestinal epithelium. Therefore, in states of negative calcium balance due to reduced vitamin D action, the compensatory mechanism of enhanced renal calcium reabsorption is compromised, despite elevated PTH levels (i.e., secondary hyperparathyroidism). In bone tissue, high PTH stimulates the production of receptor activator of nuclear factor κB ligand (RANKL) by osteoblasts; however, bone resorption is not significantly increased because VDR signaling in osteoclasts is impaired. Vitamin D activity in osteoclasts is likely necessary for full induction of osteoclastogenesis. The cumulative outcome of these processes is hypocalcemia, which leads to impaired bone matrix mineralization characteristic of osteomalacia (^75^).

### Recommended daily intake of vitamin D

In 2011, the IOM/NAM established dietary reference intakes for vitamin D for the general population (**Table 6**). They defined the minimum daily nutritional intake required to prevent vitamin D deficiency, which is characterized by serum 25-hydroxyvitamin D [25(OH)D] levels below 20 ng/mL (^76^). Thirteen years after the release of the IOM/NAM report, the Endocrine Society continues to endorse these recommendations, specifically for individuals aged 19-74 years (^77^).

**Table 6:** Dietary vitamin D recommendations for preventing hypovitaminosis D. No specific intake recommendation has been established for infants under 1 year of age

Age group or condition	Recommended daily intake	Tolerable upper intake level
1-3 years	600 IU	2500 IU
4-8 years	600 IU	3000 IU
9-70 years	600 IU	4000 IU
>70 years	800 IU	4000 IU
Pregnant and lactating women	600 IU	4000 IU

However, certain at-risk populations may benefit from achieving serum 25(OH)D levels above 30 ng/mL, which requires vitamin D intake exceeding the amounts recommended by the IOM/NAM. This need is highlighted in a joint position statement from the Brazilian Society of Endocrinology and Metabolism and the Brazilian Society of Clinical Pathology and Laboratory Medicine (^78^). Older adults are especially susceptible to vitamin D deficiency, a condition associated with increased risks of falls, secondary hyperparathyroidism, and fractures.

Based on several studies that evaluated clinical outcomes related to serum 25(OH)D levels (e.g., PTH suppression, fracture incidence, sarcopenia, muscle weakness, and risk of fall) the position statement supports a target 25(OH)D range of 30-60 ng/mL in individuals over 65 years of age to mitigate the adverse health outcomes linked to serum levels below 30 ng/mL. Other clinical conditions may also benefit from maintaining serum 25(OH)D within this range, including osteoporosis, osteomalacia, osteogenesis imperfecta, hyperparathyroidism, sarcopenia, recurrent falls, CKD, malabsorption syndromes, liver failure, anorexia nervosa, and cancer (^78^).

The Endocrine Society adheres to the IOM/NAM vitamin D intake recommendations for individuals aged 19-74 years. However, the two organizations differ in their approach for persons aged 75 years and older. The Endocrine Society recommends empirical vitamin D supplementation for healthy individuals aged 75 years and above, based on evidence suggesting a reduced risk of mortality. For this demographic, the recommended average supplemental dose is 900 IU daily (^77^).

### Brazilian scenario of vitamin D intake

Vitamin D possesses unique characteristics compared to other nutrients discussed in this paper. Its supply to the body is partly provided through dietary intake of cholecalciferol, obtained mainly from animal sources, and, to a lesser extent, ergocalciferol, derived predominantly from fungi, algae, and plants. Both forms serve as substrates for the hepatic enzyme 25-hydroxylase, which converts them into 25-hydroxyvitamin D (25(OH)D), a serum marker that reflects the body’s vitamin D stores. However, dietary intake contributes only about 20% of the total supply. Unlike other nutrients addressed here, the primary source of vitamin D is endogenous cutaneous synthesis of cholecalciferol through sun exposure, accounting for approximately 80% of the substrate required for 25(OH)D production (^79^). Thus, vitamin D status is influenced by both dietary intake and the degree of sun exposure.

The typical Brazilian diet is not rich in vitamin D, as foods with substantial amounts of cholecalciferol or ergocalciferol are limited (^80^). Two Brazilian surveys assessed nutrient intake, including vitamin D, in a combined sample of 77,493 individuals. The average daily vitamin D intake among adolescents, adults, and older adults varied by 2.0-2.4 µg/day (80-96 IU), 1.7-2.2 µg/day (68-88 IU), and 1.5-2.0 µg/day (60-80 IU), respectively. Among all nutrients evaluated, vitamin D had the highest prevalence of inadequate intake (99.4-100%) (^81^). **Table 7** summarizes the vitamin D content of the primary dietary sources of this nutrient (^82^).

**Table 7:** Vitamin D content per serving size

Food	Serving size	Vitamin D content
Sun-dried mushrooms	100 g	1600 IU ergocalciferol
Fresh mushrooms	100 g	100 IU ergocalciferol
Cod liver oil	1 Tablespoon	1360 IU cholecalciferol
Wild salmon	100 g	600-1000 IU cholecalciferol
Farmed salmon	100 g	100-250 IU cholecalciferol
Canned sardines	100 g	300 IU cholecalciferol
Canned tuna	100 g	230 IU cholecalciferol
Egg yolk	1 Unit	20 IU cholecalciferol
Cooked beef liver	100 g	15 IU cholecalciferol

### Evidence from meta-analyses and systematic reviews

A systematic review and network meta-analysis underlying the Endocrine Society’s osteoporosis guidelines evaluated the effects of various interventions on fracture risk. Compared to placebo, the combination of vitamin D and calcium resulted in a 19% relative risk reduction in hip fractures. For non-vertebral fractures, vitamin D alone reduced the risk by 56% compared to placebo (^83^,^84^).

Another meta-analysis found that combined vitamin D and calcium supplementation reduced the risk of hip fractures by 16-33%, and the risk of any fractures by 5-19%. However, variations in study populations, vitamin D dosages, and methods within this and other meta-analyses prevent definitive conclusions (^85^).

Bouillon and cols. reviewed randomized clinical trial data regarding the impact of vitamin D supplementation on a range of outcomes. The most substantial bone health benefits were observed in individuals with vitamin D deficiency. Conversely, administering high intermittent doses of vitamin D to sufficient individuals was associated with decreased BMD and an increased risk of falls and fractures (^86^).

In summary, the correlation between serum vitamin D levels and the risk of falls and fractures exhibits a U-shaped distribution, whereby both vitamin D deficiency and vitamin D excess are associated with increased risk of these outcomes. The most robust evidence for benefit from vitamin D supplementation, namely improvement in bone mineral density and reduction in falls and fractures, would be for individuals with adequate calcium intake, vitamin D doses between 800 and 2000 IU per day, elderly individuals (over 70 years of age), and individuals with vitamin D deficiency (^87^).

### Side effects and adverse events

As discussed in the physiology section, the human body has mechanisms to maintain serum calcium levels within a narrow range. Because vitamin D enhances intestinal calcium absorption, renal tubular calcium reabsorption, and bone resorption, hypercalcemia is a potential adverse effect of its use. Nevertheless, the therapeutic index of vitamin D formulations, including cholecalciferol, ergocalciferol, and calcifediol, is broad. In individuals without predisposing risk factors for hypercalcemia, serum 25(OH)D levels above 150-200 ng/mL are generally necessary to induce toxicity. This effect is partially mediated by vitamin D catabolic enzymes, which protect against intoxication (^88^).

Cases of exogenous vitamin D intoxication have been reported following ingestion of cholecalciferol, ergocalciferol, calcifediol, calcitriol, vitamin D analogs, and even topical application of vitamin D analogs for psoriasis (e.g., calcipotriol) (^88^). Among currently available vitamin D formulations, the 1α-hydroxylated compounds (calcitriol, alfacalcidol, and eldecalcitol) carry higher risks of hypercalcemia, hypercalciuria, and ectopic calcification. Their use is therefore limited to specific indications such as hypoparathyroidism and renal osteodystrophy. However, most reported cases of vitamin D intoxication since 2010 have been related to inappropriate prescribing of cholecalciferol, unauthorized high-dose cholecalciferol prescriptions, and the use of unregulated products (^79^).

The duration of vitamin D intoxication correlates with the half-life and lipophilicity of the compound involved. The half-lives of cholecalciferol, calcifediol, and calcitriol are approximately 2-3 months, 2-3 weeks, and 15 hours, respectively (^89^). Accordingly, intoxication with calcitriol often resolves more rapidly, whereas that caused by calcifediol or cholecalciferol can persist for weeks to months (^88^). Clinical manifestations of vitamin D intoxication are diverse, primarily related to hypercalcemia as detailed in the “Calcium” section.

The management of vitamin D intoxication involves restricting the intake of calcium-rich foods (limiting consumption to 400 mg/day or less) and vitamin D, avoiding sun exposure or using sunscreen when exposure cannot be avoided, increasing fluid intake, and reducing the consumption of oxalate-containing foods (^90^). These initial measures may not always suffice to resolve symptoms and hypercalcemia associated with vitamin D intoxication. Additional interventions, as detailed in **Table 8**, should be implemented according to the severity of the condition (^91^).

**Table 8:** Management strategies for vitamin D intoxication.

Intervention	Mechanism of action	Dose	Onset of action	Duration of effect	Comments
0.9% Sodium chloride	Corrects hypovolemia (primary treatment goal). Increases urinary calcium excretion	4-6 L/day (200-300 mL/hour)	Hours	During infusion	Caution in elderly patients and/or those with acute kidney injury and/or heart failure
Loop diuretics	Increases urinary calcium excretion	40-160 mg/day	Hours	During treatment	Initiate only after correction of hypovolemia. Early use may worsen hypercalcemia
Glucocorticoids	Decrease intestinal calcium absorption; reduce 1,25(OH)_2_D production; activate 24-hydroxylase	Prednisone (1 mg/kg/day)	2-5 days	Days to weeks	Side effects include hyperglycemia, immunosuppression, and myopathy
Bisphosphonates	Inhibit bone resorption	Pamidronate (90 mg + 0.9% saline 250 mL); Zoledronic acid (4 mg infusion)	24-72 hours	2-4 weeks	Contraindicated in patients with a glomerular filtration rate < 35 mL/min/1.73 m^2^

GGCX: gamma-glutamyl carboxylase; VKDPs: vitamin K dependente proteins.

## VITAMIN K

### Physiological role in bone tissue

The initial association between vitamin K and skeletal biology originated from observations of bone malformations in newborns whose mothers had taken the vitamin K antagonist warfarin during pregnancy (^92^). Vitamin K refers to a family of lipophilic molecules characterized by a common 2-methyl-1,4-naphthoquinone ring structure (^92^,^93^). Among these, phylloquinone (vitamin K1) is the primary dietary source and is found in vegetables (e.g., spinach and broccoli), fruits (e.g., kiwi and avocado), and plant oils (e.g., soy and canola) (^94^). Another form, menaquinones (MK or vitamin K2), is predominantly synthesized by bacteria and is present in foods such as meat, cheese, and fermented products (^93^). Menaquinones can also be produced by the intestinal bacterial flora. The MKs are subdivided into 13 subtypes, MK-2 through MK-14; except for MK-4, all can be synthesized in the human gut (^95^). The main characteristics of the commercially available forms of vitamin K2 are summarized in **Table 9**. Vitamin K1 is mainly involved in the production of coagulation factors and can be converted into vitamin K2 by gut flora. During this conversion, some forms of MK generate menadione as an intermediate molecule (^95^).

**Table 9:** Characteristics of commercially available forms of vitamin K2

MK subtypes	Characteristics
MK-4	Short-chain MK; shorter half-life; main form of vitamin K in the human body; can be produced from vitamin K1; not produced by human gut flora
MK-7	Long-chain MK; longer half-life; found in natto (fermented soy beans), chicken, pork, beef and cheese

Source: Adapted from the literature (^95^,^98^,^99^).

As a fat-soluble vitamin, vitamin K from dietary sources requires normal pancreatic function and the presence of bile salts for intestinal absorption, after which it is transported in chylomicrons through the lymphatic system (^96^). Vitamin K1 is primarily delivered to the liver, where it mainly facilitates the carboxylation and activation of coagulation factors. The longer side chains of vitamin K2 result in greater stability, allowing these forms to circulate in the bloodstream longer than vitamin K1 (^97^).

**Figure 2:**
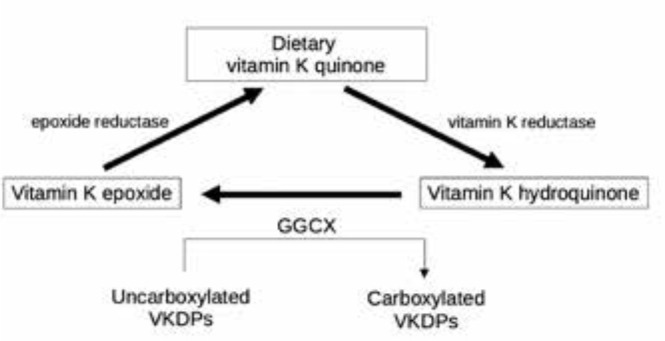
Vitamin K cycle.

Vitamin K1, MK-4, and MK-7 are also commercially produced via fermentation or chemical synthesis and are available over the counter (^100^). Despite these sources, vitamin K is efficiently recycled during the gamma-carboxylation process, which reduces the dietary requirement for this vitamin (^92^). **Figure 2** presents the vitamin K cycle.

The two main forms of vitamin K (phylloquinone and menaquinones) function as cofactors for the enzyme carboxyglutamyl carboxylase, which catalyzes the carboxylation of protein glutamic acid residues, producing active gamma-carboxyglutamic acid (Gla) residues (^101^). Among the 17 vitamin K-dependent proteins, six are involved in bone metabolism: matrix Gla protein (MGP), periostin, growth arrest-specific protein 6, protein S, and osteocalcin (OC), the most abundant non-collagenous protein in bone (^92^,^94^). By carboxylating and thereby activating MGP and OC, vitamin K plays a significant role in maintaining bone strength (**Figure 1**).

Properly carboxylated Gla residues of OC bind to calcium ions in hydroxyapatite, facilitating adequate bone mineralization, while undercarboxylated forms (ucOC) exhibit reduced affinity for calcium and hydroxyapatite (^94^). OC is specifically expressed in osteoblasts, whereas MGP is synthesized by vascular smooth muscle cells, endothelial cells, chondrocytes, osteoblasts, and osteoclasts. The gamma-carboxylated form of MGP acts as an inhibitor of calcification, preventing both vascular calcification and mineralization by osteoblasts (^94^). A recent study using MG63 cell culture demonstrated that MGP also stimulates bone formation through the upregulation of the Wnt-beta-catenin signaling pathway (^102^).

Vitamin K further influences bone remodeling through carboxylation-independent mechanisms. For example, MK-4 is involved in SXR (steroid and xenobiotic receptor)-induced gene expression and the regulation of proteins such as matrilin-2 and tsukushi, which are associated with collagen quality and quantity, as well as CD14, which modulates the genetic transcription of osteoblastic markers and osteoclastogenesis (^94^,^95^). A Japanese study also suggests that vitamin K decreases activation of the RANK pathway and increases expression of osteoprotegerin, thereby promoting bone formation and reducing bone resorption (^103^). **Table 10** lists the effects of vitamin K on bone metabolism.

**Table 10:** Effects of Vitamin K on bone metabolism

Variable	Effects
Coenzyme for the GGCX	Suppresses bone resorption Increases osteoblast genesis
Gla osteocalcin formation	Regulates osteoclast formation Enhances osteoblast collagen accumulation
Gla-rich protein and periostin production	Decreases RANKL expression Increases osteoprotegerin expression
MGP maintains bone metabolism	Induces osteoclast apoptosis SXR signaling induces osteoblast differentiation

Source: Adapted from the literature (^104^).

Both vitamin K1 and K2 deficiencies have been associated with an increased risk of fractures; however, vitamin K supplementation has not consistently demonstrated a protective effect across studies, as discussed below. One of the key challenges in conducting studies focused on bone or vascular health is the difficulty in accurately measuring plasma vitamin K levels, in addition to controlling for confounding variables such as blood lipid concentrations, the coexistence of chronic diseases, and the presence of inflammation (^105^). In this context, undercarboxylated protein levels can serve as indicators of low vitamin K concentrations and may even help to identify tissue-specific insufficiencies. For instance, levels of ucOC are considered a sensitive, indirect marker of vitamin K status in humans (^95^), while elevated levels of PIVKA-II are recognized as an indicator of hepatic vitamin K deficiency (^106^).

A meta-analysis including over 80,000 participants demonstrated that both low dietary intake and low circulating levels of vitamin K are associated with reduced BMD and an increased risk of fractures, particularly of the hip (^107^). Another investigation utilizing data from the Nurses’ Health Study found that women who consumed less than 109 mcg of vitamin K per day had a higher risk of fractures (^108^).

Furthermore, a study involving male and female participants from the Framingham Heart Study identified an association between low dietary vitamin K intake and an elevated risk of hip fractures; this study also reported a link between low intake and decreased bone density in women (^109^).

### Recommended daily intake of vitamin K

The recommended daily allowance of vitamin K is based on levels required to ensure normal blood coagulation (^94^). The Food and Agriculture Organization of the United Nations and the World Health Organization recommend 55 mcg/day for adult females and 65 mcg/day for adult males (^110^). The Food and Drug Administration recommends that adults consume 120 mcg of vitamin K daily (^111^), while the National Institutes of Health considers 120 mcg for males and 90 mcg for females to be adequate daily intakes (^112^). In Brazil, the National Health Surveillance Agency adopts 120 mcg per day as the general recommended daily value for food (^113^).

Nevertheless, studies focused on bone and vascular health suggest that higher daily intakes (exceeding even 156-250 mcg/day) may be necessary to prevent unfavorable outcomes (^114^,^115^). These findings indicate that subclinical deficiencies may impair vitamin K-dependent carboxylation before significant alterations in coagulation are observed (^116^). Thus, in situations of vitamin K deficiency, there appears to be preferential carboxylation of coagulation factors over extra-hepatic proteins (^92^).

Another notable finding is that plasma levels of K1 or MK-7 required to reduce ucOC increase with age. These requirements are higher in individuals over 70 years, a group that is also at increased risk for vitamin K-related vascular diseases, such as vascular calcifications, which have been associated with osteoporosis (^106^). The reason for this greater demand in older individuals remains to be clarified.

### Brazilian scenario of vitamin K intake

The Brazilian Institute of Geography and Statistics conducted surveys on personal food consumption in Brazil, revealing insufficient vegetable consumption, which implies low vitamin K intake among the Brazilian population (^117^). Consistent with this finding, a cross-sectional study using 3-day nutritional records from 173 healthy individuals aged 21-81 years in São Paulo, Brazil, found that the median daily vitamin K intake for adults was 99 mcg/day, while the elderly population (older than 60 years) consumed 104 mcg/day (^118^).

In addition to dietary intake, the vitamin K content in vegetables depends on cultivation conditions. A study conducted by the School of Pharmaceutical Sciences of the University of São Paulo demonstrated significant variation in vitamin K concentrations between products grown in Brazil and those planted in the United States. For example, kale and arugula cultivated in Brazilian soil contain 245.52 and 259.11 mcg/100g, respectively, while their American counterparts contain 817 and 108.60 mcg/100g, respectively (^119^).

### Evidence from meta-analyses and systematic reviews

Although vitamin K deficiency has been substantially associated with reduced BMD and increased fracture risk, vitamin K supplementation has not demonstrated clear benefits or definitive evidence for preventing these outcomes. Variations in dosage, treatment duration, and forms of supplementation (K1, MK-4, MK-7) across studies likely contribute to the heterogeneity of results. Furthermore, a recent meta-analysis revealed that the anti-fracture effect of vitamin K was only observed in long-term observational studies with follow-up durations exceeding 10 years, indicating that follow-up duration is also a relevant factor (^107^). Nevertheless, vitamin K is widely prescribed in Japan as an approved medication for osteoporosis (^95^).

### Vitamin K effects on bone mineral density

Studies about the effects of vitamin K, alone or in combination, on bone health have been published since the 60’s. Since then, several randomized clinical trials (RCTs) using either vitamin K1 or vitamin K2 supplements have been conducted, however as discussed earlier, the results are still conflicting. Hu and cols. (^120^) conducted a meta-analysis with 10 RCTs that compared the effects of vitamin K taken with calcium to control groups taking only vitamin K, or only calcium or a placebo. Six trials were performed using vitamin K1 with doses ranging from 30mcg to 5mg per day, and 7 studies used vitamin K2, from 90 mcg to 45 mg. The meta-analysis included 1,346 participants who were followed for 6 months to 4 years, and they concluded that the combination was associated with increased lumbar BMD and reduced levels of ucOC. Other meta-analyses reached less conclusive results, like the one published by Huang and cols., which included 19 RCTs and 6759 postmenopausal women taking vitamin K2 supplements, whether MK-4 (1.5 to 90 mg/day) or MK-7 (100 to 360 mcg/day). They observed significant improvements in lumbar BMD among Japanese patients with osteoporosis; however, no significant changes were found in the non-osteoporotic group (^121^). There are also meta-analyses that did not observe significant changes in BMD, like the one conducted by Salma and cols. which incorporated studies from Asia, North America, Europe, and Africa, using vitamin K1 (100 mcg to 5 mg) or K2 (180 mcg to 45 mg) for 6-36 months, either as monotherapy or in combination (^122^).

### Vitamin K effects on fractures

Few RCTs included fractures as an endpoint. In the meta-analysis conducted by Huang and cols. (^121^), the pooled analysis showed no significant differences in fracture incidence between patients who received and did not receive K2, although a subsequent sensitivity analysis demonstrated a significant benefit favoring vitamin K2 supplementation. Salma and cols. (^122^) described a decrease in the overall fracture risk in the group that received supplementation with vitamin K, mainly K2 forms in higher dosages, despite showing minimal impact on BMD, with higher doses of K2 forms exhibiting a more pronounced effect.

### Vitamin K and bisphosphonates

There are only few studies exploring the association of vitamin K with bisphosphonate treatments. Moore and colleagues (^123^) conducted a trial involving 105 postmenopausal women with osteoporosis and low baseline vitamin K1 levels, all of whom were already receiving oral bisphosphonates, calcium, and/or vitamin D. Participants received either 1 mg/day of vitamin K1, 45 mg/day of MK-4, or placebo for 18 months. No significant differences in BMD were observed among the groups; however, vitamin K1 administration led to significant changes in bone turnover markers and hip geometry parameters, as assessed by hip structural analysis software (^123^). Kasukawa and cols. (^124^) evaluated the addition of vitamin K2 to risedronate in 101 postmenopausal women with osteoporosis. They found no difference in fracture incidence after 12 months with K2 use, but patients who experienced vertebral fractures had significantly higher levels of ucOC when treated with risedronate alone. **Table 11** summarizes the types and dosages of vitamin K used in the above-mentioned studies.

**Table 11:** Vitamin K1 and vitamin K2 dosages and observed outcomes in the above-mentioned RCTs

	Dosages	Effects on BMD
Philoquinone (K1)	30 mcg - 10 mg	Increase in BMD, decrease in ucOC when used with calcium
Menatetrenone (MK-4)	1,5 mg - 90 mg	Increase in BMD in osteoporotic patients; reduced overall fracture risk
Menaquinone-7 (MK-7)	100 mcg - 10 mg	Increase in BMD in osteoporotic patients; reduced overall fracture risk

Source: Adapted from literature (^120^-^122^).

### Side effects and adverse events

There is no known toxicity associated with dietary vitamin K. The synthetic derivative of vitamin K1, phytadione, is commercially available and can be administered orally, intramuscularly, subcutaneously, or intravenously. It is indicated for the treatment of coagulation disorders due to vitamin K deficiency. Synthetic vitamin K2 is also available for oral supplementation as MK. Vitamin K toxicity is extremely rare, but may result in hemolytic anemia, hyperbilirubinemia, and jaundice (^125^).

## MAGNESIUM

### Physiological role in bone tissue

Magnesium is an important cation in the human body, facilitating numerous essential enzymatic reactions (^126^-^128^). Approximately 60% of the body’s total magnesium is stored in the bones, with about 30% of this pool available to regulate blood magnesium levels (^127^-^131^). Dietary magnesium is absorbed into the bloodstream primarily in the ileum through passive paracellular mechanisms dependent on claudins 2 and 12, as well as active transcellular mechanisms dependent on transient receptor potential cation channel subfamily M members 6 and 7 (TRPM6 and TRPM7). Calcitriol (the active metabolite of vitamin D) and inulin (a non-absorbable oligosaccharide synthesized by certain plants) can enhance intestinal magnesium absorption (^126^,^127^,^130^-^132^). Absorption is also influenced by factors such as estrogen, intestinal lumen pH, FGF-23, PTH, insulin, epidermal growth factor (EGF), and the intestinal microbiota (^130^). (**Figure 1**).

Renally, 95-97% of filtered magnesium is reabsorbed in the thick ascending limb of the loop of Henle, a process dependent on claudins 16, 19, and 10b, and in the distal convoluted tubule, through TRPM6 and TRPM7. Several factors stimulate renal magnesium reabsorption, including PTH, calcitriol, insulin, metabolic alkalosis, EGF, inulin, sodium-glucose cotransporter 2 inhibitors, amiloride, and triamterene (^126^,^130^,^132^,^133^). Conversely, reabsorption is inhibited by alcohol, insulin resistance, hypercalcemia, metabolic acidosis, hypokalemia, hypophosphatemia, thiazide and loop diuretics, calcineurin inhibitors, cisplatin, cetuximab, erlotinib, and various genetic disorders (^126^,^130^,^133^).

Plasma magnesium homeostasis is maintained by the balance between intestinal absorption, bone storage, and renal excretion (^130^). As magnesium is predominantly intracellular, only about 1% resides in plasma; thus, serum measurements may not accurately reflect total body magnesium stores (^128^,^130^,^132^). Most clinical laboratories define the normal serum magnesium as 1.7-2.4 mg/dL (0.7-1.0 mmol/L). Potential laboratory interferences include hypoalbuminemia, sample hemolysis, and the use of anticoagulants such as ethylenediaminetetraacetic acid (^130^).

Hypomagnesemia may result from inadequate dietary intake, alcoholism, gastrointestinal malabsorption, intestinal dysbiosis, type 2 diabetes, and the use of proton pump inhibitors, diuretics, H_2_ blockers, antihistamines, antibiotics, antivirals, antiepileptics, immunosuppressants, or chemotherapy agents (^126^,^130^,^134^). Hypomagnesemia is typically asymptomatic; however, potential manifestations include muscle weakness, cramps, and fatigue. Severe deficiency may lead to seizures and arrhythmias (^126^,^130^).

In cases of moderate or severe hypomagnesemia, both PTH levels and renal calcium reabsorption decrease, resulting in hypocalcemia (^129^,^130^,^134^,^135^). Mild hypomagnesemia, in contrast, can stimulate PTH secretion (^133^,^135^). When hypermagnesemia reduces PTH levels and induces hypercalciuria, it also leads to hypocalcemia (^129^,^133^,^135^). Both hypoand hypermagnesemia appear to be detrimental to bone health (^131^).

Magnesium serves as a cofactor for alkaline phosphatase, which is indispensable for bone matrix synthesis and mineralization. Magnesium also promotes bone formation by stimulating osteoblast proliferation. Furthermore, it is necessary for enzymes involved in activating vitamin D and participates in maintaining calcium homeostasis (^126^-^130^,^132^,^134^). Osteoclastogenesis inducer IκB kinase is regulated by magnesium; thus, deficiency can facilitate osteoclast survival, proliferation, and differentiation (^126^).

Hypomagnesemia acts by reducing osteoblast numbers, triggering local inflammation in bone tissue, and enhancing osteoclast differentiation and activity. It also impairs the activation of vitamin D, whose active form is important for promoting osteoblast differentiation and proliferation, as well as for inhibiting osteoclast generation. Collectively, hypomagnesemia increases osteoclast activity while decreasing osteoblast activity, ultimately leading to elevated bone resorption (^126^-^130^,^132^,^134^).

An *in vitro* study by Mammoli and cols. (^131^) demonstrated that hypermagnesemia significantly reduces the expression of osteogenic genes *RUNX2* and *COL1A1* and is associated with diminished matrix calcification. Moreover, hypermagnesemia impaired protein kinase activity, which further promoted osteoclast differentiation and inhibited osteoblast differentiation. Overall, this study suggests that hypermagnesemia inhibits osteoblast differentiation while promoting osteoclast differentiation, thereby enhancing bone resorption.

### Recommended daily intake of magnesium

The recommended daily allowance (RDA) for magnesium is 300-320 mg/day for adult women, 350-430 mg/day for adult men (^126^-^128^,^130^,^134^), and 350-360 mg/day for pregnant women (^126^).

The adequate intake (AI), the estimated average requirement (EAR), the recommended daily allowance (RDA), and the tolerable upper intake level (UL) for magnesium are detailed in **Table 12**. In pregnant women, the EAR for magnesium is between 290 and 335 mg/day and the RDA is between 350 and 400 mg/day. In lactating women, the EAR is between 255 and 300 mg/day and the RDA is between 310 and 360mg/day (^136^).

**Table 12:** Magnesium intake recommendations

Age	mg/day	
0 to 6 months	30	AI
7 to 12 months	75	
1 to 3 years	65	EAR
	80	RDA
	65	UL of supplementary magnesium
4 to 8 years	110	EAR
	130	RDA
	110	UL of supplementary magnesium
9 to 13 years	200	EAR
	240	RDA
Male		
14 to 18 years	340	EAR RDA
	410	
19 to 30 years	330	EAR RDA
	400	
≥ 31 years	350	EAR RDA
	420	
Female		
14 to 18 years	300	EAR RDA
	360	
19 to 30 years	255	EAR RDA
	310	
≥ 31 years	265	EAR RDA
	320	
> 8 years	350	UL of supplementary magnesium

Adequate intake (AI), estimated average requirement (EAR), recommended daily allowance (RDA), tolerable upper intake level (UL). Source: Adapted from the literature (^136^).

The richest dietary sources of magnesium include legumes, vegetables, nuts, seeds, and whole grains (**Table 13**) (^126^-^128^,^130^,^134^). Magnesium bioavailability can be reduced by food refining processes, cooking, and exposure to the herbicide glyphosate. Magnesium content in water varies substantially depending on the source (^126^,^127^). Organic magnesium salts (e.g., glycinate, lactate, citrate, gluconate, and aspartate) are absorbed more efficiently than inorganic salts (e.g., oxide, chloride, and carbonate) (^130^).

**Table 13:** Examples of commonly consumed foods and their approximate magnesium content per portion

Some examples of foods rich in magnesium	Approximate magnesium content (mg)
Spinach, cooked, 200 g	150
Pasta, whole grain, boiled, 200 g	90
Seeds (pumpkin seeds, sunflower seeds, and/or pine nuts), one tablespoon	55
Mixed nuts, one handful	55
Peanuts, one handful	55
Red kidney beans, boiled, 100 g	50
Banana, a middle-sized	40
Bread, whole grain, one slice	25

Source: Adapted from the literature (^134^).

Low dietary magnesium intake can lead to hypomagnesemia, which treatment involves increasing magnesium intake through dietary sources or supplementation. In cases where oral supplementation fails to correct hypomagnesemia, intravenous magnesium replacement is indicated. Pharmacologic interventions such as amiloride, triamterene, inulin, and sodium-glucose cotransporter 2 inhibitors may also be employed (^130^).

### Brazilian scenario of magnesium intake

The prevalence of hypomagnesemia is 3-10% in the global population, 10-30% among individuals with type 2 diabetes, 10-60% in hospitalized patients, and exceeds 65% in patients admitted to intensive care units (^130^).

Pinheiro and cols. conducted the BRAZOS study analyzing mineral and vitamin intake in a Brazilian population over the age of 40 using a 24-hour dietary recall questionnaire. 80% of the participants had an inadequate dietary magnesium intake. Magnesium intake was measured in individuals with and without fragility fractures (**Table 14**), and the difference was statistically significant for women but not for men (**Table 14**). As for regional disparities in magnesium intake, the only statistically significant finding was a higher magnesium intake among participants with fragility fractures in the northern region of Brazil (^30^).

**Table 14:** Magnesium observed intake in Brazilian studies

			Mean observed intake
Pinheiro and cols.	With history of fragility fracture	Men	244
		Women	196
	Without history of fragility fracture	Men	201
		Women	189
Hermes Sales and cols.	Men	**Years old**	
		14-18	225.2
		19-30	256.8
		31-50	249.4
		51-70	239.1
		≥ 71	237.1
	Women	14-18	199.2
		19-30	196.4
		31-50	193.7
		51-70	188.3
		≥ 71	184.0
Verly-Jr and cols.	**2008-2009**		
	Men	Adolescents	285.8
		Adults	310.0
		Elderly	285.3
	Women	Adolescents	244.3
		Adults	240.8
		Elderly	232.4
	**2017-2018**		
	Men	Adolescents	273.3
		Adults	304.7
		Elderly	275.7
	Women	Adolescents	229.7
		Adults	232.8
		Elderly	225.5
Rodrigues and cols.	Adolescents		243.91

Source: Adapted from the literature (^30^,^31^,^81^,^138^).

In northeastern Brazil, a study involving pregnant women reported a prevalence of magnesium deficiency (defined as <1.8 mg/dL) of approximately 50% (^137^). Another study, Hermes Sales and cols., found that over 80% of the São Paulo population has inadequate dietary magnesium intake (**Table 14**) (^138^).

The study by Varly-Jr and cols. consisted of two food records covering the last 30 days of 32,749 individuals in two consecutive years (2008 and 2009) and two 24-hour dietary recalls of 44,744 individuals in another two consecutive years (2017 and 2018). Regarding magnesium intake, a deficiency of more than 50% was found in adolescents and more than 60% in adults and the elderly (**Table 14**) (^81^).

Rodrigues et. al conducted a cross-sectional survey of adolescents aged 15-18 years living in São Paulo revealed that more than 70% of participants did not meet the recommended daily magnesium intake (**Table 14**) (^31^).

### Evidence from meta-analyses and systematic reviews

Dominguez and cols. (^132^) conducted a meta-analysis investigating the association between serum magnesium levels and fracture incidence. Their findings revealed a statistically significant association between low serum magnesium levels and an increased risk of fractures (relative risk = 1,579; 95% confidence interval = 1,216-2,051; *p*-value = 0.001). Although the authors considered the overall number of participants adequate, they noted several limitations incluinding the small number of eligible studies, the fact that all those eligible studies were observational, and the fact that half of those studies involved patients undergoing hemodialysis (^132^).

Another meta-analysis by Groenendijk and cols. (^134^) examined the relationship between magnesium intake (from supplements and food) and various bone parameters, including BMD (total body, lumbar spine, femoral neck, and hip), bone turnover markers, and fracture risk (total and hip). The only parameter suitable for meta-analysis was the association between magnesium intake and hip BMD, for which a positive association was identified (p < 0.05). Limitations of this Groenendijk and cols. study also consist of the small number of eligible studies included and the fact that most of them were observational.

Sari and cols.’s meta-analysis considered nine randomized controlled trials, but no significant correlation was found between oral magnesium supplementation and fracture incidence. Limitations include the fact that the follow-up time of eligible studies varied considerably and there was considerable heterogeneity between the effect sizes across eligible studies (^139^).

There is a meta-analysis of randomized clinical trials in hemodialysis patients, which analyzes the correlation between magnesium supplementation and serum calcium, phosphorus, and PTH levels and carotid intima-media thickness. However, no research has been conducted on the relationship between magnesium supplementation and bone parameters or fractures (^140^).

Reviews by Fatima and cols., Rondanelli and cols. and Liu and cols. discuss the interaction of magnesium with bone physiology agents (see section “Physiological role in bone tissue”) and suggest that hypomagnesemia may have a potentially negative effect on bone health (^126^,^128^,^129^). However, to date, there are only a few meta-analyses on magnesium and its relationship with bone parameters or fractures. There is no solid evidence from randomized controlled clinical trials to support magnesium supplementation for fracture prevention.

### Side effects and adverse events

Hypermagnesemia is a rare condition that predominantly occurs as a result of kidney failure, particularly in association with the use of magnesium-retaining medications (^128^,^130^,^133^). Hypermagnesemia is usually asymptomatic; however, possible symptoms include diarrhea, nausea, and fatigue (^130^,^133^). In severe cases, symptoms may include lethargy, hypotension, urinary retention, respiratory depression, and bradycardia (^133^). An Italian study reported a prevalence of hypermagnesemia of 1.78% among hospitalized patients, defining hypermagnesemia as a serum level > 3.8 mg/dL (^141^).

The treatment of hypermagnesemia involves discontinuing all exogenous sources of magnesium. In severe cases, treatment options include administration of calcium gluconate, loop diuretics, and intravenous normal saline. Hemodialysis is reserved for cases of severe hypermagnesemia that do not respond to other measures, particularly in patients with advanced renal dysfunction (^133^).

In conclusion, magnesium is a cation that the human body cannot synthesize and must obtain through dietary sources. Its potential importance for bone health highlights the need for adequate intake and appropriate clinical management, as well as further studies on the subject, particularly longitudinal studies.

## PHOSPHORUS

### Physiology and homeostasis of phosphorus

Phosphorus is a highly reactive chemical element that combines with oxygen under physiological conditions. In the human body, phosphorus predominantly exists as organic or inorganic phosphate (Pi) (^142^). Its most common natural form is pentavalent, combined with oxygen as the phosphate ion (PO_4_^3-^) (^136^). Phosphorus is an essential component of the bone mineral matrix. In adults, approximately 85% (around 700 g) of total body phosphorus is stored in bone tissue (^143^). Bones and teeth serve as the primary reservoirs of phosphorus in humans, where it is present as calcium phosphate crystals (apatite); the remainder is distributed among soft tissues and the extracellular fluid (ECF) (^144^). In soft tissues, phosphorus exists as soluble phosphate ions, and it is also present in lipids, proteins, carbohydrates, and nucleic acids in the form of ester or anhydride bonds, where it functions as a modulator of enzymatic activity (^143^).

Under physiological conditions, plasma phosphate concentration is maintained by a dynamic interplay among dietary intake (with efficient intestinal absorption), renal excretion (modulated by renal tubular reabsorption), and bone remodeling. (**Figure 1**). This regulatory system is governed by endocrine mechanisms involving FGF-23, PTH, and the active form of vitamin D, 1,25-dihydroxyvitamin D [calcitriol, 1,25(OH)_2_D] (^145^).

Phosphate is provided through the diet as either inorganic salts or organic phosphate compounds, which can be hydrolyzed by intestinal alkaline phosphatases or bacterial phytases. Inorganic phosphate is absorbed in the intestine by two primary pathways: the transcellular pathway, mediated by active transport through sodium-dependent transporters such as PiT1, PiT2, and NaPi-IIb, located in the luminal membrane of small intestinal enterocytes, and the paracellular pathway, which appears to be independent of both dietary phosphorus intake and calcitriol levels (^142^,^146^).

Approximately 15% of total body phosphorus is stored in soft tissues, primarily in the liver and skeletal muscle. Phosphate uptake by muscle is stimulated by insulin via the PiT1 and PiT2 transporters. In the liver, phosphate plays a crucial role in metabolic processes, including glycolysis and phospholipid synthesis (^142^). Skeletal muscle functions as the main storage site of phosphorus in soft tissues, mainly as adenosine triphosphate and phosphocreatine (^144^). Adequate Pi levels are essential for muscle energy metabolism (^147^), and deficiency may result in myopathy, which is reversible with phosphate supplementation, as observed in osteomalacia related to vitamin D deficiency or tumor-induced osteomalacia (^142^,^144^). Beyond its structural role, phosphate acts as a signaling molecule in bone remodeling, influencing osteoblast and osteoclast activity through local pathways involving sclerostin, RANKL, and osteoprotegerin (^142^,^148^,^149^). It is estimated that approximately 180 mg of Pi are deposited through bone formation, and an equal amount is mobilized via resorption every 24 hours (^144^).

The kidneys are the primary regulators of plasma phosphate concentration. About 80% of filtered phosphate is reabsorbed in proximal tubules via NaPi cotransporters, a process modulated by FGF-23, PTH, and calcitriol (^144^,^150^). This process depends on the GFR and the tubular maximum capacity for phosphate reabsorption (TmPi) (^151^,^152^).

The maximum tubular reabsorption rate of Pi (TmPi) can be clinically assessed using fasting blood and urine samples collected over 1-2 hours, with a mid-interval blood draw. Calculations involve formulas that incorporate plasma and urinary Pi and creatinine concentrations (^144^,^153^). The fractional excretion of Pi can also be expressed relative to creatinine clearance to evaluate urinary phosphate loss (^154^). In adults, TmPi/GFR (maximum tubular phosphate reabsorption capacity adjusted for GFR) is calculated using the Walton and Bijvoet nomogram (^155^) or the Kenny and Glen algorithm (^153^,^156^) (**Table 15**). Reference values for TmPi/GFR in adults range from 0.8 to 1.35 mmol/L of glomerular filtrate (^153^).

**Table 15:** Renal parameters for the evaluation of phosphate reabsorption and excretion

Fractional excretion of phosphate	FEPi (%) = [(Pi_u_ × Cr_p_) / (Pi_p_ × Cr_u_)] × 100
Tubular reabsorption of phosphate (TRPi)	TRP = 1 - FEPi
Tubular maximum reabsorption rate per GFR (TmPi/GFR)	If TRP < 0.86: TmP/GFR = TRP × Pi_p_ If TRP > 0.86: TmPi/GFR = α × Pi_p_ α = 0.3 × TRP / (1 - [0.8 × TRP])

Pi_u_: Urinary phosphate; Cr_p_: plasma creatinine; Pi_p_: plasma phosphate; Cr_u_: urinary creatinine.

Source: Based on the literature (^144^,^154^,^156^).

Phosphate homeostasis is regulated by an endocrine axis primarily composed of the hormones FGF-23, PTH, and 1,25(OH)_2_D. Under physiological conditions, FGF-23 is produced by osteocytes and osteoblasts in response to high phosphorus intake, elevated serum phosphate levels, and increased concentrations of 1,25(OH)_2_D (^145^). Acting on the renal proximal tubule through the FGFR-Klotho receptor complex, FGF-23 decreases the activity of sodium-phosphate cotransporters (NaPi-IIa and NaPi-IIc), thereby reducing phosphate reabsorption and enhancing urinary excretion (^144^,^153^). Additionally, FGF-23 suppresses renal 1α-hydroxylase, limiting the synthesis of 1,25(OH)₂D, and induces 24-hydroxylase, which catalyzes the degradation of both 25(OH)D and 1,25(OH)₂D, collectively reducing intestinal absorption of calcium and phosphate (^153^,^157^). Conversely, reduced extracellular phosphate inhibits FGF-23 secretion, which favors an increase in TmPi and enhances intestinal phosphate absorption, thereby contributing to the maintenance of normophosphatemia (^144^).

PTH is secreted in response to hypocalcemia and/or elevated serum phosphate. It stimulates the production of 1,25(OH)_2_D and may also induce FGF-23 secretion. In cases of hyperphosphatemia, circulating levels of both PTH and FGF-23 increase, acting on the kidneys to downregulate the expression of the NaPi-IIa and NaPi-IIc cotransporters. This process reduces phosphate reabsorption capacity and promotes phosphaturia. Conversely, during hypophosphatemia, increased synthesis of 1,25(OH)_2_D_3_ stimulates the intestinal expression of the NaPi-IIb cotransporter, enhancing the efficiency of dietary phosphate absorption (^158^).

### Recommended daily intake of phosphorus

A typical diet generally provides sufficient phosphorus, making dietary phosphate deficiency unlikely under most circumstances (^159^). Dietary phosphate exists in two primary forms: organic and inorganic (^158^). Organic phosphate from plant sources is mainly present as phytates, while animal-derived phosphate is bound to proteins. The most significant source of Pi is the addition of Pi salts as food additives (^142^).

Approximately 30% of dietary phosphorus is derived from meats (animal tissues) and vegetables, another 30% comes from dairy products, with about 70% of this being Pi, and the remaining 40% originates from phosphate added as food additives, processed foods, and dietary supplements (^144^,^160^).

Phytate (inositol hexakisphosphate), found in plants, is rich in phosphorus and is more prevalent in vegetarian diets. However, the bioavailability of phosphorus from phytate is low in humans, as plant-derived phytase is inactivated by cooking, and the human intestinal tract expresses negligible levels of endogenous phytase activity (^144^,^158^,^161^). Interestingly, the gut microbiota in individuals who consume high-phytate diets (e.g., vegetarians) is more effective in degrading phytate. This suggests that the microbiome can adapt to dietary environments and that diet may modulate bacterial metabolic activity (^161^).

In addition to naturally occurring food phosphorus, total dietary phosphorus intake is significantly influenced by phosphate-containing additives, which are widely used as preservatives and flavor enhancers in ultra-processed foods, fast foods, and soft drinks. Because these additives are rich in highly bioavailable phosphate salts and have become increasingly common, the actual percentage of absorbable phosphate has risen considerably in recent years, often exceeding dietary recommendations, particularly in industrialized nations (^158^,^162^).

The RDA for phosphorus in adults is 700 mg/day, and the EAR is 580 mg/day (^136^). The tolerable UL is 4000 mg/day (^163^), although some evidence suggests that such a high intake may contribute to the development of bone and cardiovascular diseases (^144^,^158^). **Table 16** presents the usual mean daily phosphorus intake, EAR, RDA, and UL by sex and age group.

**Table 16:** Usual mean daily phosphorus intake and dietary reference intake guidelines (EAR, RDA, and UL) by sex and age. Values are given in mg/dL

Sex and age	Usual phosphorus intake	EAR	RDA	UL
Men				
1-3	1030 ± 26.3	380	460	3000
4-8	1145 ± 27.4	405	500	3000
9-13	1321 ± 35.4	1055	1250	4000
14-18	1681 ± 61.5	1055	1250	4000
19-30	1656 ± 53.4	580	700	4000
31-50	1727 ± 25.0	580	700	4000
51-70	1492 ± 30.0	580	700	4000
≥ 71	1270 ± 27.6	580	700	3000
Women				
1-3	1030 ± 26.3	380	460	3000
4-8	1145 ± 27.4	405	500	3000
9-13	1176 ± 57.5	1055	1250	4000
14-18	1067 ± 29.8	1055	1250	4000
19-30	1120 ± 40.8	580	700	4000
31-50	1197 ± 25.0	580	700	4000
51-70	1106 ± 34.0	580	700	4000
≥ 71	985 ± 28.8	580	700	3000

EAR: Estimated average requirement; RDA: recommended dietary allowance; UL: tolerable upper intake level. Source: Adapted from the literature (^10^,^163^-^166^).

The RDA for phosphorus in pregnant and lactating women up to 12 months postpartum is 1250 mg for those aged 14-18 years and 700 mg for those aged 19-50 years (^164^,^166^). Among infants, dietary phosphorus intake varies based on the food source. Phosphorus bioavailability is highest in human milk (85-90%), intermediate in cow’s milk (72%), and lowest in soy-based formulas (~59%) due to the presence of phytic acid. Although human milk contains less phosphorus, infant formulas compensate for lower absorption rates by providing higher concentrations of phosphorus, calcium, and other minerals, thereby resulting in greater overall mineral absorption (^136^). Nevertheless, the lower phosphorus intake from human milk may be advantageous, as it helps decrease fecal pH and inhibits the proliferation of pathogenic microorganisms in the distal gut, while also safeguarding the immature renal system of the newborn (^136^,^167^-^169^).

### Brazilian scenario of phosphorus intake

A Brazilian analysis using data from the National Dietary Survey (2008-2009; 2017-2018) evaluated phosphorus intake across different age and sex groups: 1,094.6-1,081.9 mg for male adolescents, 1,167.0-1,153.4 mg for adult men, and 1,036.6-977.1 mg for older men; and 977.1-924.6 mg, 928.9-878.9 mg, and 877.3-811.2 mg for female adolescents, adults, and older adults, respectively. Despite these relatively high mean intakes, the study found that more than half of Brazilian adolescents had phosphorus intake below the EAR, reflecting the higher physiological requirements during adolescence and explaining the prevalence of inadequacy in this age group (^81^).

Over the past decade, phosphorus consumption in Brazil has risen. The proportion of ultra-processed foods in total energy intake increased from 18.7% to 19.7% between 2008 and 2018, alongside higher sugar, fat, and sodium intake and reduced consumption of nutrient-dense foods (^170^). Many of these products contain phosphate-based additives with nearly complete intestinal absorption, substantially increasing total phosphorus exposure and potentially disturbing mineral homeostasis (^171^, ^172^).

In a cross-sectional study among manufacturing workers in Northeastern Brazil, frequent consumption of processed meat products - such as sausages, mortadella, hamburgers, and nuggets containing mechanically separated meat - was associated with higher intakes of energy, fat, sodium, and phosphorus, and lower intake of minimally processed foods (^173^). Among adolescents, regular intake of soft drinks, fast foods, and other ultra-processed products was linked to poorer overall diet quality and greater exposure to phosphate additives (^174^).

This pattern aligns with evidence from the NOVA food classification system, which associates higher consumption of ultra-processed foods with poorer overall nutrient profiles, characterized by increased exposure to industrial additives and reduced intake of nutrient-dense foods (^170^). Collectively, these findings indicate that excessive phosphorus intake from additive-rich, ultra-processed foods represents an emerging nutritional and metabolic concern in the Brazilian population (^171^). In light of the increasing share of ultra-processed foods in the Brazilian diet, enhanced monitoring of phosphate additives should be considered a nutritional priority. This recommendation is consistent with recent European frameworks, such as the *Nordic Nutrition Recommendations 2023*, which emphasize the high bioavailability of phosphorus additives and the current lack of comprehensive data on their intake and long-term health implications (^175^).

### Scientific evidence

Phosphorus deficiency is rare in the individuals with normal renal function and adequate dietary intake. Homeostatic mechanisms involving renal excretion and endocrine regulation (FGF-23, PTH, and vitamin D) maintain serum phosphate within narrow physiological range. Deficiency typically occurs only in specific pathological contexts, such as chronic malnutrition, severe gastrointestinal disorders, or inherited renal phosphate-wasting syndromes (^145^,^176^). However, acute increases in inorganic phosphate intake, particularly from additives, can transiently elevate plasma phosphate and alter calcium-phosphate and PTH dynamics even in healthy adults (^177^).

In randomized cross-over trials involving healthy young adults, short-term loading with inorganic phosphate for five days led to significant increases in plasma phosphate and FGF-23 concentrations, even in the context of preserved renal function (^178^). Similarly, controlled experimental studies demonstrate that short-term ingestion of phosphorus-based food additives increases circulating FGF-23, osteopontin, and osteocalcin, while decreasing sclerostin, representing early endocrine and skeletal responses even in individuals with normal kidney function (^179^).

The balance between calcium and phosphorus intake plays a fundamental role in bone mineralization and remodeling, as both minerals are major components of the hydroxyapatite matrix and tightly regulated by endocrine and renal mechanisms (^180^). Experimental and clinical evidence indicates that diets with a disproportionate calcium-to-phosphorus (Ca:P) ratio, particularly those with excessive phosphorus relative to calcium, can disrupt mineral homeostasis, stimulate secondary hyperparathyroidism, and compromise skeletal integrity (^180^,^181^). Experimental studies demonstrate that high phosphorus intake can impair skeletal microarchitecture. In rats with normal renal function, Fernández-Villabrille and cols. demonstrated that chronically elevated dietary phosphorus intake induced significant bone deterioration, with a particular impact on trabecular bone microstructure (^182^). Rizzoli and cols. emphasize that high phosphorus intake combined with insufficient calcium increases parathyroid hormone concentrations and bone resorption, a condition frequently observed in Westernized diets rich in processed foods (^180^). Likewise, Takeda and cols. highlighted that the widespread use of phosphate additives markedly elevates dietary phosphorus exposure, lowering the Ca:P ratio, and poses risks to bone mineralization (^181^). These findings indicate that maintaining an adequate Ca:P ratio, through limiting additive-derived phosphorus and ensuring sufficient calcium intake, constitutes a nutritional strategy to preserve bone structure and mineral homeostasis (^180^-^182^).

Phosphate excess also contributes to cardiovascular injury through direct effects on vascular smooth muscle cells (VSMCs), thereby driving medial arterial calcification and loss of elasticity (^183^). In parallel, phosphate retention induces a compensatory increase in FGF-23, which acts directly on cardiomyocytes and promotes pathological left ventricular hypertrophy independent of kidney function (^184^). Collectively, these mechanisms show how phosphate excess and FGF-23 dysregulation act synergistically to drive vascular and cardiac damage (^183^-^184^). **Table 17** summarizes the main dietary sources of phosphorus and highlights the potential health risks associated with excessive intake.

**Table 17:** Main dietary sources of phosphorus and potential risks of excessive intake

Category	Examples/Main foods	Bioavailability/Chemical form	Evidence and potential health risks
Unprocessed and minimally processed foods	Milk, yogurt, white cheese, eggs, fresh meat, fish	Organic phosphate bound to proteins; moderate bioavailability (approximately 40-60 %)	Adequate intake balanced with calcium maintains bone and mineral homeostasis.
Plant sources (legumes, nuts and whole grains)	Beans, lentils, soybeans, peanuts, cashews, oats, whole wheat, brown rice	Organic phosphate in phytates; low bioavailability (< 40%)	High plant intake rarely causes excess phosphorus (P); lower absorption than animal sources.
Processed meats and sausages	Ham, sausage, mortadella, nuggets, industrial hamburgers	Inorganic phosphate additives; high bioavailability (approximately 80-100%)	Major contributors to excessive phosphorus intake; inorganic additives are rapidly absorbed and stimulate FGF-23 secretion, bone remodeling, and vascular calcification.
Cola-type soft drinks & phosphoric acid beverages	Cola, energy drinks, industrial soft drinks	Inorganic phosphate additives; high bioavailability (approximately 80-100%)	Observational data show lower bone mineral density in women with frequent cola intake, although findings were inconsistent across populations.
Ultra-processed foods	Sliced bread, processed cheese, condensed milk, fast food, frozen meals	Mix of highly bioavailable phosphate additives	Primary dietary source of inorganic phosphate additives. Highly absorbable phosphorus increases serum phosphate, PTH, and FGF-23. Chronic intake promotes endothelial dysfunction, vascular calcification, and bone remodeling alterations.

Source: Adapted from literature (^142^,^176^,^185^,^186^).

### Side effects and adverse events

#### Phosphorus disorders

In adults, normal plasma Pi levels range from 2.5 to 4.5 mg/dL (0.81-1.45 mmol/L) (**Table 18**) (^144^,^150^). A diurnal variation in plasma phosphate concentration is observed, ranging from 0.6 to 1.0 mg/dL, with a nadir occurring around 11:00 am and a peak at approximately 12:30 am (^187^,^188^). In children, phosphate concentrations are higher due to a greater maximum tubular reabsorption capacity (TmPi), which decreases with age until adult reference values are reached (^144^). In adults, serum phosphate levels tend to decline with advancing age, reflecting changes in renal tubular phosphate reabsorption (^189^).

**Table 18:** Reference ranges for serum phosphate

Age (years)	Males			Females	
	mmol/L	mg/dL		mmol/L	mg/dL
1-4	1.39-1.74	4.3-5.4		1.39-1.74	4.3-5.4
5-13	1.19-1.74	3.7-5.4		1.29-1.68	4.0-5.2
14-15	1.13-1.52	3.5-5.3		1.13-1.58	3.5-4.9
16-17	1.0-1.52	3.1-4.7		1.0-1.52	3.1-4.7
≥18	0.81-1.45	2.5-4.5		0.81-1.45	2.5-4.5

Reference values for patients under 12 months of age have not been established.

Source: Adapted from the literature (^145^,^190^).

Proper regulation of phosphorus metabolism is essential throughout life, as imbalances can result in hyperphosphatemia or hypophosphatemia, which are frequently associated with severe clinical manifestations (^144^,^145^). Chronic hypophosphatemia may lead to musculoskeletal disorders, such as delayed bone mineralization (rickets in children and osteomalacia in adults), as well as proximal muscle weakness (^144^). Additional manifestations include respiratory muscle weakness, bone pain and loss, neuropathy, cardiac dysfunction, and hematologic and neurologic abnormalities (^142^,^191^). Persistent hypophosphatemia affects multiple systems, including skeletal, muscular, and dental, ultimately compromising physical function and quality of life (^145^). Conversely, acute hyperphosphatemia may be associated with hypocalcemia; if chronic and uncontrolled, it can lead to vascular and soft tissue calcifications, increasing the risk of necrotic skin lesions, cardiac arrhythmias, and phosphate crystal deposition in joints, tendons, and eyes (^192^). **Table 19** summarizes clinical conditions and pathophysiological mechanisms associated with phosphorus disorders.

**Table 19:** Clinical conditions and pathophysiological mechanisms associated with serum phosphorus disorders

Chronic Hypophosphatemia	
Clinical Disorder	Pathophysiological Mechanism
Primary or secondary hyperparathyroidism	Reduced TmPi (tubular maximum phosphate reabsorption); elevated PTH
Phosphorus deficiency or use of phosphate binders	Reduced dietary intake and bioavailability
X-linked hypophosphatemic rickets/osteomalacia (PHEX mutation)	Reduced TmPi; elevated FGF-23
Tumor-induced osteomalacia (FGF-23-producing tumor)	
Autosomal dominant hypophosphatemic rickets/ osteomalacia (FGF-23 mutation)	
Autosomal recessive hypophosphatemic rickets/ osteomalacia (DMP1 mutation, ENPP1 mutation)	
Intravenous iron administration	
Fibrous dysplasia/McCune-Albright syndrome (GNAS1 gain of function)	
Hypercalciuric hypophosphatemic nephrolithiasis	Reduced TmPi; decreased NaPi2a and NHERF1 expression
Hyperphosphatemia	
Clinical Disorder	Pathophysiological Mechanism
Chronic kidney disease	Decreased glomerular filtration rate (GFR); phosphate retention
Hypoparathyroidism	Decreased PTH; increased TmPi
Familial hyperphosphatemic tumoral calcinosis	Genetic mutations in FGF-23, Klotho, or Galnt3
Rhabdomyolysis	Extensive muscle injury with massive phosphate release

NaPi2a: Sodium-dependent Phosphate Transporter 2A; NHERF1: Na^+^/H^+^ Exchanger Regulatory Factor 1.

Source: Adapted from the literature (^144^,^145^).

Hypophosphatemia is defined as a serum phosphate level below 2.5 mg/dL in adults and below 4.0 mg/dL in children (^179^). It can be classified as moderate (0.3-0.65 mmol/L [1-2.5 mg/dL]) or severe (< 0.3 mmol/L [< 1.0 mg/dL]). In contrast, hyperphosphatemia is defined by plasma phosphate levels > 1.46 mmol/L (4.5 mg/dL) and is considered severe when levels exceed 14 mg/dL (^187^,^188^).

Mild to moderate hypophosphatemia can be managed with oral supplementation of sodium phosphate and potassium phosphate (^187^,^193^), which are typically compounded in specialized pharmacies in Brazil due to their limited availability in standard pharmacies. The therapeutic dose should aim for at least 1 g/day (15 mg/kg) and may be increased to up to 3 g/day in cases of severe phosphate depletion (^187^,^193^). Common adverse effects include diarrhea and gastrointestinal irritation, especially when doses exceed 1 g. To improve tolerability, the total daily dose should be divided into three or four administrations (^187^).

In cases of severe hypophosphatemia or when clinically significant symptoms are present (e.g., cardiac, respiratory, or neurological manifestations), intravenous phosphate replacement is indicated. It should be initiated in the emergency setting with continuous monitoring. Intravenous administration should be performed using either 0.9% sodium chloride or 5% dextrose solutions; lactated Ringer’s solution should be avoided due to the risk of calcium phosphate precipitation (^187^,^193^). Serum phosphate, calcium, and magnesium levels should be monitored every 6 hours during parenteral therapy to detect complications, including rebound hyperphosphatemia, hypocalcemia, and hypomagnesemia (^187^). A commonly used protocol involves infusing 4.5 mmol/h over 3 hours, with a maximum dose of 90 mmol over 24 hours. Alternatively, 0.08 mmol/kg may be administered over 6 hours for acute cases and 0.16 mmol/kg over 6 hours for chronic depletion states, which are generally more resistant to correction (^187^).

Management of hyperphosphatemia should focus on treating the underlying cause, such as intravenous volume resuscitation in cases of rhabdomyolysis or tumor lysis syndrome. In patients with CKD and mild, asymptomatic hyperphosphatemia, dietary phosphate restriction and intestinal phosphate binders containing aluminum, calcium, or magnesium are recommended. In severe cases, hemodialysis or peritoneal dialysis provides rapid and effective correction of hyperphosphatemia and associated symptomatic hypocalcemia (^187^).

## CONCLUSION

Taken together, these micronutrients each play distinct yet interconnected roles in bone health. Evidence from randomized controlled trials supports the combined use of calcium and vitamin D in older adults and individuals with deficiency, as this strategy effectively reduces fracture risk. In contrast, the roles of vitamin K and magnesium remain promising but inconsistent, emphasizing the need for further well-designed randomized trials to clarify their clinical benefits. Phosphorus deficiency is uncommon in healthy adults; however, excessive intake - particularly from processed foods - may contribute to adverse metabolic and vascular outcomes. Whenever possible, dietary sources should be prioritized, reserving supplementation for populations at risk or with confirmed deficiencies. A concise overview of the supporting evidence and clinical considerations is provided in **Table 20**.

**Table 20:** Summary of evidence, clinical recommendations, and adverse effects of key micronutrients in bone health

Nutrient	Strength of Evidence	Clinical Recommendations	Adverse Effects/Risks
Calcium	High (multiple meta-analyses and RCTs)	Supplementation recommended for individuals with low intake or osteoporosis risk, preferably in combination with vitamin D	Gastrointestinal discomfort, hypercalcemia, nephrolithiasis, possible increased cardiovascular risk in those with adequate dietary intake
Vitamin D	High (consistent RCTs and meta-analyses)	Combined calcium + vitamin D supplementation reduces fracture risk in older adults and deficient populations; monitoring advised in high-dose regimens	Hypercalcemia, hypercalciuria, toxicity with inappropriate use
Vitamin K	Moderate to low (heterogeneous clinical trials)	Evidence suggests potential skeletal benefits, but current data are inconsistent; further RCTs required before recommending routine supplementation	Generally safe; possible interaction with some anticoagulants (e.g., warfarin)
Magnesium	Moderate to low (observational and limited interventional studies)	Maintain adequate dietary intake; supplementation may be beneficial in individuals with low intake or deficiency	Diarrhea, gastrointestinal discomfort; toxicity rare, usually in renal impairment
Phosphorus	Moderate to low (strong physiological rationale; limited clinical data)	Deficiency is uncommon; supplementation reserved for specific conditions (e.g., hypophosphatemia). Excess intake from processed foods should be avoided	Hyperphosphatemia, vascular calcification, secondary hyperparathyroidism, especially in renal disease

## Data Availability

datasets related to this article will be available upon request to the corresponding author.
