# erratum

**DOI:** 10.20945/2359-4292-2026-0026

**Published:** 2026-03-19

In the article **“Supplements for bone health”**, published in Archives of Endocrinology and Metabolism, DOI: 10.20945/2359-4292-2025-0374:

Page 4, Table 2

Where it reads:

Fruits – Banana – 221.99 mg/100 g

It should read:

**Fruits – Banana – 4.29 mg/100 g**

